# ERK3 regulates TDP2-mediated DNA damage response and chemoresistance in lung cancer cells

**DOI:** 10.18632/oncotarget.6682

**Published:** 2015-12-19

**Authors:** Ka Bian, Naveen Reddy Muppani, Lobna Elkhadragy, Wei Wang, Cheng Zhang, Tenghui Chen, Sungyun Jung, Ole Morten Seternes, Weiwen Long

**Affiliations:** ^1^ Department of Biochemistry and Molecular Biology, Wright State University, Dayton, OH, USA; ^2^ Department of Otorhinolaryngology, Tangdu Hospital, The Fourth Military Medical University, Xi'an, China; ^3^ Department of Molecular and Cellular Biology, Baylor College of Medicine, Houston, TX, USA; ^4^ Department of Bioinformatics and Computational Biology, The University of Texas MD Anderson Cancer Center, Houston, TX, USA; ^5^ Department of Pharmacy, University of Tromsoe, N-Tromsoe, Norway

**Keywords:** ERK3, TDP2, Top2 inhibitors, DNA damage, chemoresistance

## Abstract

Posttranslational modifications (PTMs), such as phosphorylation and ubiquitination, play critical regulatory roles in the assembly of DNA damage response proteins on the DNA damage site and their activities in DNA damage repair. Tyrosyl DNA phosphodiesterase 2 (TDP2) repairs Topoisomerase 2 (Top2)-linked DNA damage, thereby protecting cancer cells against Top2 inhibitors-induced growth inhibition and cell death. The regulation of TDP2 activity by post-translational modifications in DNA repair, however, remains unclear. In the current study, we have found that ERK3, an atypical MAPK, phosphorylates TDP2 at S60 and regulates TDP2's phosphodiesterase activity, thereby cooperatively protecting lung cancer cells against Top2 inhibitors-induced DNA damage and growth inhibition. As such, our study revealed a post-translational regulation of TDP2 activity and discovered a new role of ERK3 in increasing cancer cells’ DNA damage response and chemoresistance to Top2 inhibitors.

## INTRODUCTION

Tyrosyl DNA phosphodiesterase 2 (TDP2), originally designated as TRAF and TNF receptor-associated protein (TTRAP), [1] and ETS1-associated protein II (EAPII), [2], was recently revealed as a (and the first) human 5′-tyrosyl DNA phosphodiesterase [3]. It hydrolyzes the phosphotyrosyl bond between topoisomerase 2 (Top2) and DNA and repairs Top2-linked DNA double-strand break that can be greatly enhanced by Top2 inhibitors such as doxorubicin and etoposide [3-5]. As such, TDP2 protects cells against Top2 inhibitors-induced DNA damage, growth inhibition and apoptosis, thereby conferring chemoresistance. In line with this, TDP2 has been shown to be overexpressed in lung cancer [6, 7] and knockdown of TDP2 remarkably increases lung cancer cells’ sensitivity to Top2 inhibitors, underscoring the importance of targeting TDP2 enzyme in clinic treatment. Posttranslational modifications (PTMs) such as phosphorylation and ubiquitination play critical roles in DNA damage repair by regulating the recruitment of DNA damage repair proteins, their enzymatic activities and the assembly and stability of the DNA damage repair complex [8]. For example, the activity and functions of tyrosyl DNA phosphodiesterase 1 (TDP1), a 3′-tyrosyl DNA phosphodiesterase, are tightly regulated by several PTMs, including ATM-dependent phosphorylation [9, 10] and sumoylation [11]. By contrast to TDP1, the posttranslational regulation of TDP2 activity remains unknown.

Extracellular signal-regulated kinase 3 (ERK3) is an atypical mitogen-activated protein kinase (MAPK) [12, 13]. In contrast with the well-studied conventional MAPKs, such as ERK1/2, much less is known regarding the activation and regulation of ERK3 signaling [14]. A gain of interests on ERK3 has been seen owing to recent important findings on its physiological and pathological roles in lung. ERK3 knockout mice display intrauterine growth restriction and neonatal lethality primarily due to defect in functional differentiation of the lung [15]. Our recent study revealed that ERK3 is upregulated in human lung tumours and promotes lung cancer cell invasiveness both *in vitro* and *in vivo* [16]. Our knowledge of ERK3 kinase's targets is still limited. To date, MAPK-activated protein kinase 5 (MK5), [17-18], steroid receptor coactivator-3 (SRC-3), [16] and Borgs [19] are the only known substrates of ERK3.

In the current study, we identified TDP2 as a novel substrate of ERK3. ERK3 phosphorylates TDP2 and promotes its phosphodiesterase activity, thereby upregualting TDP2-mediated DNA damage response and desensitizing lung cancer cells to Top2 inhibitor-induced growth inhibition. To our knowledge, this is the first report regarding the post-translational regualtion of TDP2 activity and the role for ERK3 in inceasing DNA damage response and drug resistance.

## RESULTS

### ERK3 interacts with TDP2

**Figure 1:**
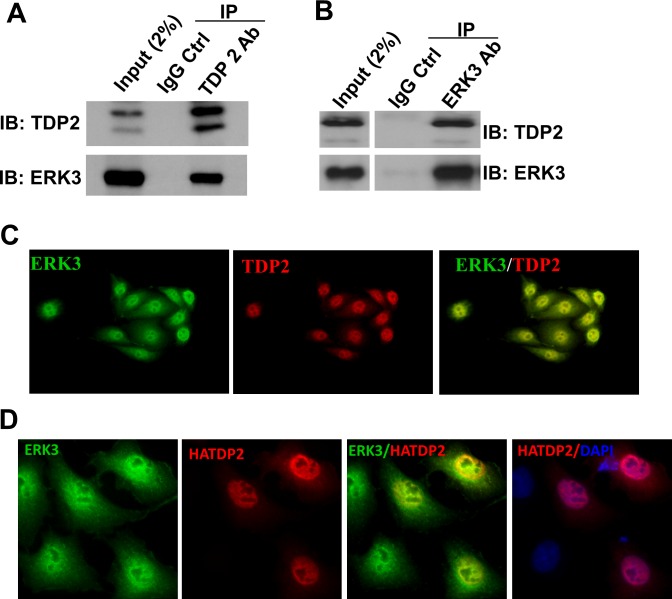
ERK3 interacts with TDP2 **A.** and **B.** The interaction between ERK3 and TDP2 in H460 cells was analyzed by co-immunoprecipitation (co-IP) using a TDP2 Ab **A.**, an ERK3 Ab **B.**, or a corresponding control IgG (IgG Ctrl), followed by Western blotting. IB: immuno-blot. **C.** Immunofluorescent staining of endogenous ERK3 and TDP2 in H460 cells. Overlapping of immunofluorescent signals between ERK3 and TDP2 is reflected by the yellow immunofluorescence resulting from the merge of the images. Magnification: 200 X. **D.** A549 cells were transfected with TDP2 with a HA tag at the N-terminus (HATDP2). Two days post-transfection, endogenous ERK3 proteins and exogenously expressed TDP2 proteins (HATDP2) were immuno-labelled with an ERK3 antibody and a HA antibody, respectively. Overlapping of immunofluorescent signals between ERK3 and TDP2 is reflected by the yellow immunofluorescence resulting from the merge of the images. DNA was stained with DAPI (Blue) for showing the nucleus. Magnification: 200 X.

We attempted to elucidate ERK3 signaling by starting the identification of ERK3 interacting proteins. For this purpose, endogenous ERK3 protein complex in H460 lung cancer cells was analyzed by immunoprecipitation-mass spectrometry (IP-MS) following the procedures described in our previous study [20]. Among protein candidates identified (data not shown), TDP2, as a Tyrosyl DNA phosphodiesterase, caught our attention in particular. Interestingly, TDP2 was also identified as an interacting partner of ERK3 by Yeast-two-hybrid screening in a large-scale interactome analysis of cellular signalling proteins [21]. The interaction between ERK3 and TDP2 was validated by co-immunoprecipitation using a TDP2 antibody (Figure 1A) or an ERK3 antibody (Figure 1B) followed by Western blotting, and further verified by immunofluorescent double staining of ERK3 and TDP2 (Figure 1C and Figure 1D). Of note, ERK3 and TDP2 primarily co-localize in the nucleus.

### ERK3 and TDP2 cooperatively protects lung cancer cells against Top2 inhibitors-induced DNA damage

**Figure 2:**
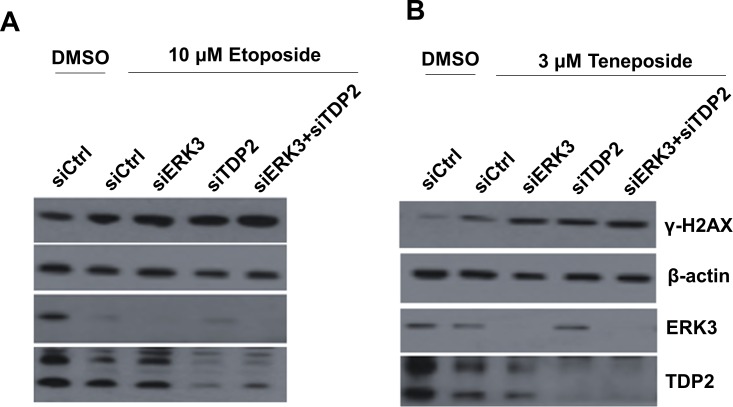
Knockdown of ERK3 and TDP2 increases H2AX phosphorylation (γ-H2AX) induced by Top2 inhibitors in H460 cells Cells were transfected with siRNAs as indicated: non-targeting control siRNA (siCtrl), siRNA specifically targeting ERK3 (siERK3), or siRNA specifically targeting TDP2 (siTDP2). 48 hrs post-siRNA transfection, cells were treated with either etoposide, teneposide or DMSO vehicle control for 1.5 hrs. Cells were then harvested and protein lysates were analyzed by Western blotting using each specific antibody as indicated.

**Figure 3:**
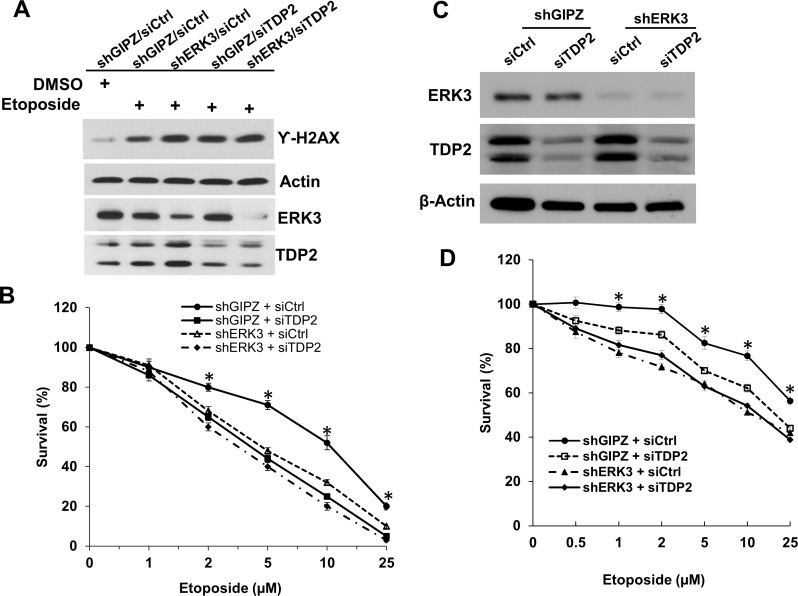
Knockdown of ERK3 and TDP2 sensitizes cancer cells to etoposide-induced cell growth inhibition A549 **A.** and **B.** and H460 **C.** and **D.** cell pools stably expressing either non-targeting shGIPZ or shERK3 were treated with either non-targeting control siRNA (siCtrl) or siRNA specifically targeting TDP2 (siTDP2) for 48 hrs. **A.** A549 cells were then treated with etoposide (10 μM) for 1.5 hrs, followed by cell lysis and Western blot analysis of γ-H2AX and protein levels of ERK3 and TDP2. **B.** A549 cells were treated with etoposide at different concentrations for 2 days. Cell survival was then analyzed by MTS cell proliferation assay. Data was presented as survival % (normalized to the sample with vehicle treatment (0 μM of etoposide). Values are means ± s.e of three separate experiments. “*” indicates significant difference (*P* < 0.01). **C.** Knockdown of ERK3 and TDP2 in H460 cells (before etoposide treatment) was verified by Western Blotting. **D.** H460 cell survival assay upon the treatment with etoposide, following the experiment approach as shown in **B.** Values are means ± s.e of three separate experiments. “*” indicates significant difference (*P* < 0.01).

TDP2 regulates cancer cells’ response to DNA damage and growth inhibition induced by Top2 inhibitors. As TDP2 and ERK3 interact with each other and co-localize in the nucleus, we hypothesized that ERK3 regulates TDP2's activity in DNA damage response. We first tested whether ERK3 plays a similar role in protecting cells against Top2-induced DNA damage. Indeed, similar to knockdown of TDP2 (siTDP2), knockdown of ERK3 (siERK3) greatly increased H2AX phosphorylation (γ-H2AX, a marker of DNA damage) induced by either Etoposide (Figure 2A) or Teneposide (Figure 2B) in H460 lung cancer cells. Interestingly, as compared to single knockdown of either TDP2 or ERK3, simultaneous knockdown of both ERK3 and TDP2 (siERK3 + siTDP2), did not lead to further significant increase of γ-H2AX, suggesting that TDP2 and ERK3 cooperatively regulate Top2 inhibitors-induced DNA damage in a non-additive manner. Similarly, knock down of ERK3 (shERK3/siCtrl, Figure 3A), TDP2 (shGIPZ/siTDP2, Figure 3A) or both (shERK3/siTDP2, Figure 3A) increased γ-H2AX in A549 lung cancer cells treated with etoposide. Of note, we found that in line with previous findings, lung cancer cell lines display highly differential response to Top2 inhibitor. H157 lung cell line shows high basal level of γ-H2AX, and etoposide treatment (even at the concentration of 20 μM) did not clearly alter γ-H2AX level (Figure S1A). In H1395 cells, however, γ-H2AX was undetectable even under the conditions with both TDP2 knockdown and etoposide treatment (Figure S1B). Although etoposide treatment did increase γ-H2AX level in H1437 lung cancer cells, knockdown of TDP2 did not show obvious effect (Figure S1C). As such, in our current study, we focused on investigating the roles of TDP2 and ERK3 in DNA damage response in H460 and A549 cell lines.

### ERK3 and TDP2 protect cells against Top2 inhibitors-induced growth inhibition and apoptosis

Cells would undergo growth inhibition and apoptosis if DNA damage is not repaired upon the treatment with Top2 inhibitors [22]. We thus determined the effects of knocking down ERK3 and TDP2 on Top2 inhibitor-induced cell growth inhibition and apoptosis. As expected, in comparison with non-targeting shRNA/siRNA control (shGIPZ + siCtrl), knockdown of TDP2 in both A549 (shGIPZ + siTDP2, Figure 3B) and H460 (shGIPZ + siTDP2, Figure 3D) sensitized cancer cells to etoposide-induced cell growth inhibition. Knockdown of ERK3 (shERK3 + siCtrl; Figure 3B and 3D) also greatly enhanced etoposide-induced growth inhibition in both cell lines. Simultaneous knockdown of ERK3 and TDP2 (shERK3+siTDP2), as compared to single knockdown, did not significantly further enhance cancer cells’ sensitivity to etoposide, suggesting ERK3 and TDP2 cooperatively regulate the response of cancer cells to top2 inhibitor-induced DNA damage and growth inhibition.

**Figure 4:**
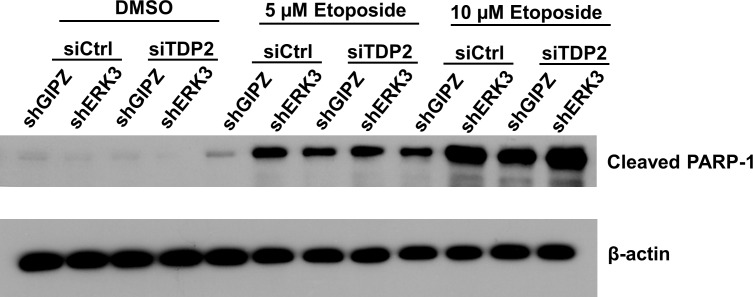
Knockdown of ERK3 and TDP2 promotes PARP-1 cleavage induced by etoposide H460 cell pools stably expressing either non-targeting shGIPZ or shERK3 were transiently transfected with either non-targeting control siRNA (siCtrl) or siRNA specifically targeting TDP2 (siTDP2). 24 hrs post-siRNA transfection, cells were treated with either DMSO or etoposide for 2 days. PARP-1 cleavage was analyzed by Western blotting.

We also examined the effects of ERK3 and TDP2 in etoposide-induced PARP-1 cleavage, a marker of topoisomerase inhibitor-induced apoptosis [23]. As shown in Figure 4, knockdown of either ERK3 (siCtrl/shERK3), TDP2 (siTDP2/shGIPZ) or both (siTDP2/shERK3) greatly increased the levels of cleaved PARP-1, suggesting that ERK3 and TDP2 also protects cancer cells against etoposide-induced apoptosis.

### ERK3 phosphorylates TDP2 *in vitro* and promotes TDP2's phosphodiesterase activity

**Figure 5:**
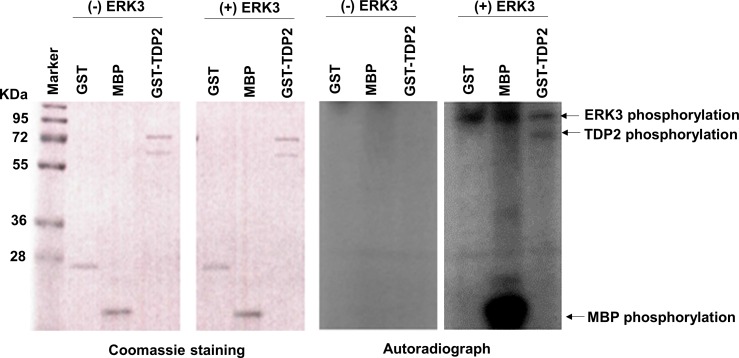
ERK3 phosphorylates TDP2 *in vitro* 1 μg of Purified GST, Myelin basic protein (MBP), or GST-TDP2 protein (Coomassie staining, left two panels) was incubated with (+) or without (−) ERK3 protein kinase (30 ng). ERK3 autophosphorylation and phosphorylations of TDP2 and MBP (indicated by arrows) are seen in the autoradiograph of the fourth panel. GST and MBP serve as a negative target and a positive target of ERK3, respectively. Please note that: 1. While ERK3 autophosphorylation was seen in autoradiograph (Panel 4), ERK3 protein (∼100 Kd) was not shown by coomassie staining (Panel 2) as only 30 ng of ERK3 protein was used in each reaction; 2. GST-TDP2 proteins were expressed as two forms and were shown as two bands by coomassie staining (Panels 1 and 2). However, only the upper band was phosphorylated by ERK3 *in vitro* (indicated by an arrow in Panel 4).

**Figure 6:**
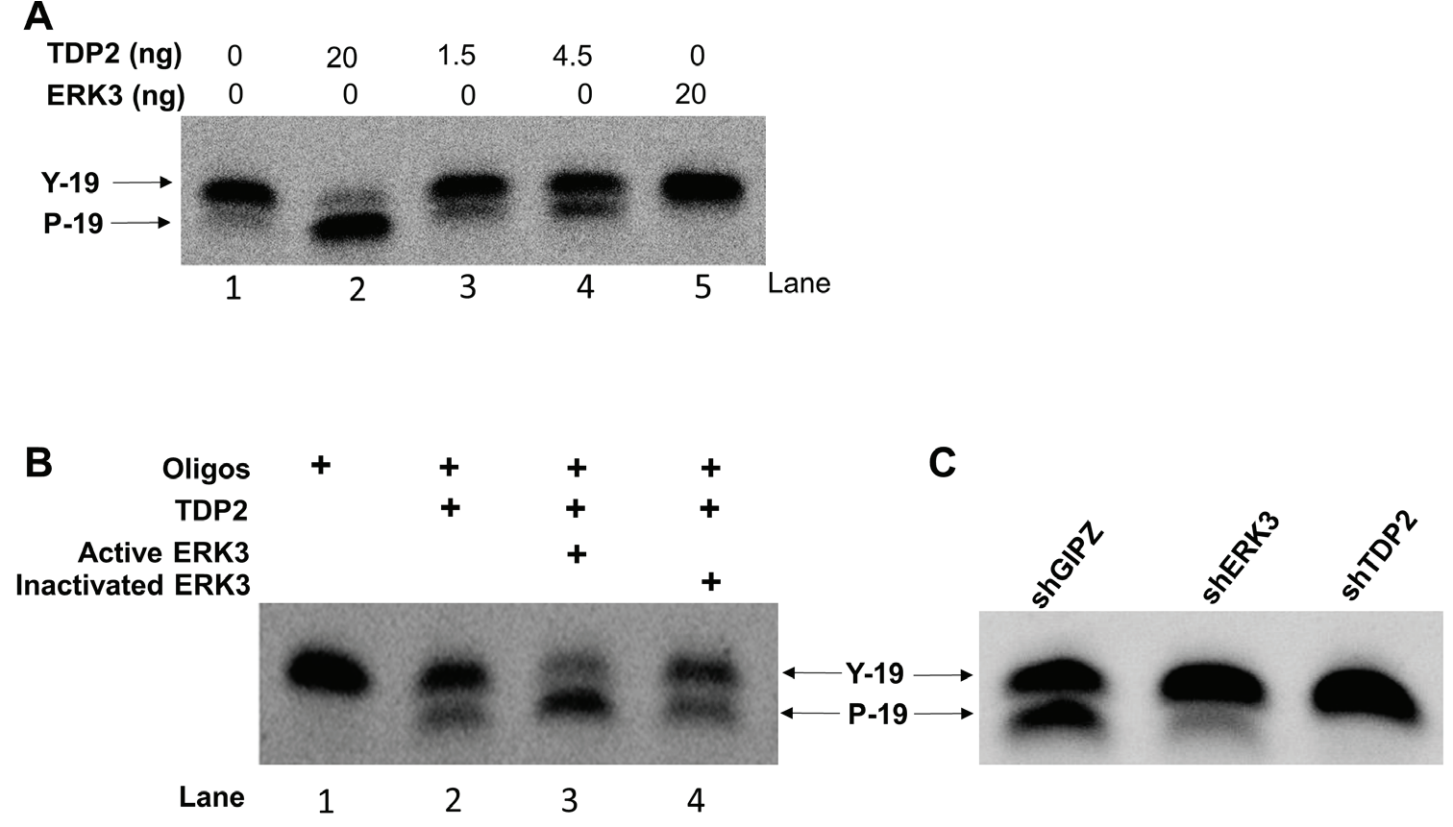
ERK3 stimulates TDP2 phosphodiesterase activity **A.** Optimization of *in vitro* tyrosyl DNA phosphodiesterase assay with the addition of different amounts of purified TDP2 protein or ERK3 protein only. **B.** 20 ng of active ERK3 kinase or heat-inactivated ERK3 protein was added together with TDP2 (1.5 ng) in phosphodiesterase assay. **C.** Tyrosyl DNA phosphodiesterase assays using whole cell lysate of H460 stably expressing either shGIPZ, shERK3 or shTDP2. Y-19: non-cleaved oligonucleotide substrate linked with a phosphotyrosine at 5′. P-19: oligonucleotides with the cleavage of tyrosyl group and with a phosphor group remained at 5′. The cleavage of oligonucleotide substrate by TDP2 phosphodiesterase was indicated by the mobility shift of oligonucleotides.

As ERK3 is a protein kinase and interacts with TDP2, we hypothesised that ERK3 phosphorylates TDP2 and regulates TDP2 tyrosyl DNA phosphodiesterase activity, thereby protecting cells against Top2-inhibitor-induced DNA damage and growth inhibition. To test whether TDP2 is phosphorylated by ERK3, we performed *in vitro* ERK3 kinase assay. As shown in Figure 5, ERK3 phosphorylates TDP2 *in vitro*. We then tested the effect of ERK3 on TDP2 phosphodiesterase activity. Purified TDP2 protein exhibited tyrosyl DNA phosphodiesterase activity *in vitro* (indicated by the presence of cleaved band) in a concentration dependent manner, whereas EKR3 did not (Lane 5, Figure 6A). To determine the effect of ERK3 on TDP2 enzymatic activity, we chose to add a dose of 1.5 ng of TDP2, at which TDP2 elicits minimum phosphodiesterase activity (Figure 6A). Importantly, addition of ERK3 to the reaction greatly enhanced the cleavage of the tyrosyl phosphorylated oligonucleotides (Lane 3, Figure 6B), whereas ERK3 inactivated by boiling did not show stimulating effect (Lane 4, Figure 6B). To determine the regulation of TDP2 phosphodiesterase activity by ERK3 in cultured cells, total cell lysates were prepared from H460 cells stably expressing shCtrl, shERK3 or shTDP2 and were analysed for TDP2 tyrosyl phosphodiesterase activity. As expected, depletion of TDP2 (shTDP2) abolished tyrosyl phosphodiesterase activity in H460 cells (Figure 6C). Importantly, depletion of ERK3 (shERK3) also greatly reduced tyrosyl phosphodiesterase activity in cultured cells (Figure 6C).

**Figure 7:**
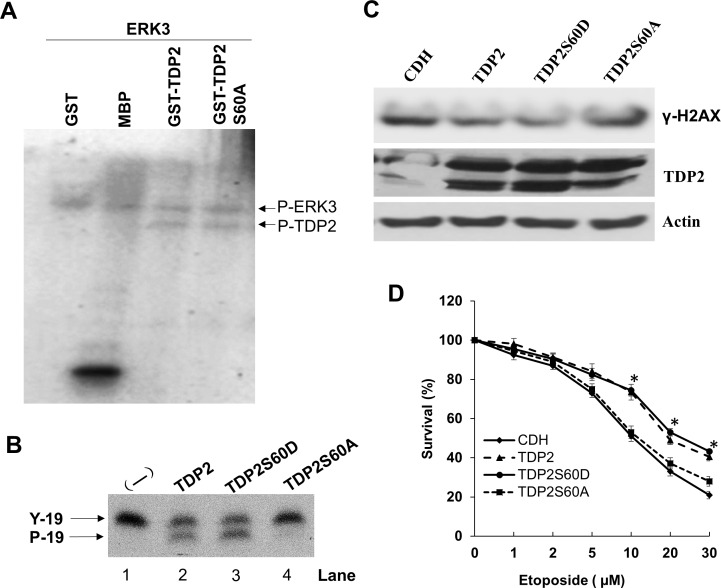
Serine 60 is required for TDP2's phosphodiesterase activity and is important for TDP2-mediated DNA repair induced by etoposide **A.** *In vitro* ERK3 kinase assay using purified GST, MBP, GST-TDP2 or GST-TDP2S60A protein as a substrate. **B.** *In vitro* TDP2 tyrosyl DNA phosphodiesterase assay. 4.5 ng purified protein of TDP2, TDP2S60D or TDP2S60A was added to the reaction. Lane 1: a reaction containing oligonucleotide substrate but without the addition of TDP2 enzyme. **C.** and **D.** A549 cells with stable expression of shRNA specifically targeting 3′-UTR region of TDP2 mRNA (shTDP2) were transduced with lentiviruses expressing either the pCDH vector (CDH), TDP2, TDP2S60D or TDP2S60A. 48 hrs post-lentiviral transduction, cells were then treated with etoposide either at the concentration of 10 μm for 1.5 hrs for analyzing TDP2 expression and γ-H2AX by Western blotting **C.**, or at different concentrations for 2 days for analyzing drug sensitivity by MTS cell proliferation assay **D.** For drug sensitivity analysis, data was presented as survival % (normalized to the sample with vehicle treatment (0 μM of etoposide). Values are means ± s.e of three separate experiments. “*” indicates significant difference (*P* < 0.005) between CDH (or TDP2S60A) and TDP2 (or TDP2S60D).

To identify the specific phosphorylation site(s) of TDP2, phosphorylated GST-TDP2 by ERK3 *in vitro* was analyzed by LC-Mass spectrometry. S60 of TDP2 was identified to be a phosphorylation site targeted by ERK3 (Supplemental Table 1). To verify this, Serine 60 was mutated to alanine and *in vitro* ERK3 kinase assay was performed using purified TDP2S60A as a substrate. However, TDP2S60A was phosphorylated by ERK3 at the equivalent level to that of wild type TDP2 (Figure 7A). This might be that there is ERK3 target site (s) other than S60 in TDP2 and this site could not be detected in the LC-MS analysis. Nevertheless, we tested the role of S60 in TDP2's tyrosyl DNA phosphodiesterase activity. Interestingly, mutation of S60 to alanine (TDP2S60A) almost abolished TDP2's phosphodiesterase activity *in vitro* (Lane 4, Figure 7B), whereas TDP2S60D (Lane 3, Figure 7B) has slightly increased activity as compared to wild type TDP2. We then examined the importance of S60 in TDP2's role in regulating DNA damage response to etoposide treatment in cultured cells. As compared to that of CDH vector control, exogenous expression of TDP2 or TDP2S60D in A549 cells with stable knockdown of endogenous TDP2 greatly decreased etoposide-induced γ-H2AX level (Figure 7C) but significantly increased cell survival (Figure 7D) in the presence of etoposide treatment, whereas reintroduction of TDP2S60A had little effect. Taken together, these results demonstrate that ERK3 positively regulates TDP2's tyrosyl DNA phosphodiesterase activity, possibly through phosphorylating S60 and thereby protecting cells against Top2-inhibitor-induced DNA damage and growth inhibition.

## DISCUSSION

ERK3 (an atypical MAPK) differs from ERK1/2 (conventional MPAKs) in regard to the activation and functions. ERK3 is constitutively phosphorylated at its activation motif and is primarily localized in the nucleus [24]. ERK3 has been shown to phosphorylate MK5 and SRC-3 [16-18]. Phosphorylation of MK5 by ERK3 leads to the translocation of MK5 from the nucleus to the cytoplasm, while phosphorylation of SRC-3 by ERK3 stimulates the formation of a transcriptional complex containing SRC-3, CBP and PEA-3 and the subsequent expression of matrix metalloprotease genes. In this study, we identified TDP2 as a new target of ERK3 and they work together to protect cancer cells against Top2 inhibitors-induced DNA damage and growth inhibition. Interestingly, although there is no previous report specifically about the role of ERK3 in DNA damage repair and drug resistance, ERK3 was shown to be upregulated in MCF-7 cells with resistance to doxorubicin (another Top2 inhibitor) as compared to the parental MCF-7 cells that are sensitive to this drug [25].

PTMs play essential roles in the process of DNA damage repair by regulating the stability, the recruitment to the DNA damage site and the activities of DNA damage response proteins [8]. For example, TDP1 is modified by phosphorylation (at S81), SUMOylation and PARylation, all of which are important for the recruitment of TDP1 to DNA damage site and its function in repairing DNA damage [22]. The posttranslational regulation of TDP2's activity, however, is virtually unknown. Our current study demonstrates that ERK3 phosphorylates TDP2 and stimulates its tyrosyl DNA phosphodiesterase activity. Structural analysis revealed that TDP2 contains an N-terminal ubiquitin-associated (UBA) domain, which may regulates TDP2 enzymatic activity through the intramolecular interaction with the c-terminal catalytic domain [26]. Interestingly, S60 of TDP2, the ERK3 target site, is located in the UAB domain and mutation of S60 to alanine abolished TDP2's phosphodiesterase activity (Figure 7B). Thus, it would be important to determine whether phosphorylation of S60 mediates this intermolecular interaction, thereby regulating TDP2 enzymatic activity. It is of note that S60A mutation did not alter ERK3-mediated TDP2 phosphorylation *in vitro*. We postulate that there is ERK3 target site (s) other than S60 in TDP2, but the phosphorylation on this site was not detected in the LC-MS analysis due to the limitation of tryptic digestion. This putative phosphorylation site may also play a role in regulating TDP2 activity, but S60 is critical so that mutation of this single residue almost abolishes TDP2's activity.

Although Top2 inhibitors, such as etoposide and doxorubicin, have been used for treating cancer patients, their clinic use is limited by drug resistance and the adverse effects including toxicity and secondary malignancy [27]. Much interest has been seen recently on investigating TDP2 as it repairs Top2-mediated DNA damage and confers cancer cells’ resistance to Top2 inhibitors [22]. Thus inhibiting TDP2 activity would sensitize cancer cells’ to Top2 inhibitors-induced growth inhibition and killing, thereby increasing the therapeutic efficacy and decreasing the toxicity of Top2 inhibitors in cancer treatment. On the basis of this therapeutic rationale, a high-throughput screening (HTS) for TDP2 inhibitors was recently carried out and toxoflavins and deazaflavins were identified as selective inhibitors of this enzyme [28]. However, the further *in vivo* study of these two inhibitors are challenged due to their redox activity and/or low cell permeability. Our finding that TDP2 activity is regulated by a phosphorylation site in the regulatory UBA domain promises an alternative strategy for targeting TDP2. In addition, ERK3, as a protein kinase, would be a potentially attractive anti-cancer target as well.

## MATERIALS AND METHODS

### Expression plasmids

pGEX-2TK-EAPII (TDP2) was provided by Dr. Runzhao Li at Emory University and was described previously [2]. pGEX-2TK-TDP2S60A harbouring a point mutation of serine 60 to alanine and pGEX-2TK-TDP2S60D harbouring a point mutation of serine 60 to aspartic acid were generated by site-directed mutagenesis using the Quick-change kit (Stratagene, La Jolla, CA) and the sequence of the resulting mutant was verified by sequencing. pCMV-HA-TDP2 was provided by Drs. Rui Gao and Yves Pommier at NCI and was described previously [5]. pGIPZ lentiviral shRNAmir expression constructs, including non-silencing negative control pGIPZ-shCtrl (RHS4346), pGIPZ-shERK3 (clone ID V3LHS_344615) and pGIPZ-shTDP2 (clone ID V2LHS_234163), were purchased from Open Biosystems. pCDH-TDP2 was generated by inserting the TDP2 fragment released from pGEX-2TK-TDP2 by Dra I/Stu I digestion into pCDH-CMV-MCS-EF1-Puro (System Biosciences) digested with Swa I. Similarly, pCDH-TDP2S60A and pCDH-TDP2S60D were generated by inserting TDP2S60A and TDP2S60D, respectively, into pCDH-CMV-MCS-EF1-Puro.

### Cell culture, transfection and treatment with Top2 inhibitors

Lung cancer cell lines H460 and A549 from American Type Culture Collection (ATCC) were maintained in RPMI 1640 medium supplemented with 10% FBS. HEK293T cells were cultured in Dulbecco's modified Eagle medium supplemented with 10% FBS. All the culture media and supplements were purchased from Life Technologies. siRNAs were transfected into the cells using TransIT-TKO Transfection Reagent (Mirus Bio Corporation) following the manufacturer's instructions. TDP2 siRNA on-target plus SMART pool and non-targeting siRNA pool were purchased from Dharmacon. The silencer select siRNAs targeting human ERK3 and the silencer negative control #1 were purchased from Ambion. Unless specifically indicated, cells were harvested for various analyses 48 hrs post-transfection. For experiments involving the treatment with Top2 inhibitors etoposide or teneposide (Sigma Aldrich), cells were treated with various concentrations of Top2 inhibitors or DMSO for different time periods as indicated in each specific experiment. Cells were then harvested for protein extraction.

### Immunoprecipitation (IP) and western blotting

Cells were lysed with EBC lysis buffer (50 mM Tris, pH 7.5, 150 mM NaCl, 0.5% NP-40, 1 mM PMSF, 1 mM Complete protease inhibitors (Roche Diagnostics) and 1 mM Phosphatase Inhibitor Cocktail III (Sigma). One mg of total protein lysate of H460 cells was used for each IP using 2 μg of TDP2 antibody (Bethyl Laboratories), ERK3 antibody (Bethyl Laboratories), or normal rabbit IgG (Bethyl Laboratories). The supernatant was precleared with 40 μl protein A/G agarose beads for 1 hr at 4°C with constant rotation. The samples were then incubated with desired antibody for 2 hrs, followed by the addition of 40 μl protein A/G agarose beads for additional 1 hr. The beads were washed three times (5 min/wash) with lysis buffer. Proteins were boiled off the beads in 2x Laemmili sample buffer and resolved on 4-15% SDS-PAGE gels (BioRad). 2% of the amount of protein supernatant for IP was loaded as the input control. Western blotting was performed by first blocking nitrocellulose membranes with 5% nonfat milk in PBS-T buffer for 30min, followed by overnight incubation with primary antibody at 4°C and 1 hr incubation with appropriate secondary antibody at room temperature. The Western blot was visualized by chemiluminescence (Amersham). Primary antibodies used in Western blotting were: anti-TDP2 (Bethyl Laboratories), anti-ERK3 (Millipore), anti-phospho-H2AX (γ-H2AX) antibody, anti-cleaved PARP (Cell Signaling Technology) and anti-β-actin (Millipore).

### Immunofluorescence

A549 cells were transfected with pCMV-HA-TDP2 with a HA tag at the N-terminus (HATDP2). Two days post-transfection, endogenous ERK3 proteins and exogenously expressed TDP2 proteins were immuno-labelled with a rabbit anti-ERK3 antibody (Millipore) and a mouse anti-HA antibody (Sigma Aldrich), respectively, following the procedures as described previously [29]. The subcellular localization of endogenous ERK3 and TDP2 was examined in H460 cells. Twenty-four hours after seeding H460 cells on cover slips in RPMI 1640 medium, cells were analysed by immunofluorescent staining using a mouse anti-ERK3 antibody (Santa Cruz Biotechnology) and a rabbit anti-TDP2 (Bethyl Laboratories). Images were captured with a Zeiss AxioVert S100 TV deconvolution microscope and a DeltaVison restoration microscopy system (Applied Precision, Inc.).

### *In vitro* ERK3 kinase assay

Active ERK3 kinase was expressed and purified in sf9 insect cells as described previously [18]. pGEX-2TK-TDP2, pGEX-2TK-TDP2S60A and pGEX-2TK-TDP2S60D were expressed in E. coli (BL21). GST-TDP2, GST-TDP2S60A and GST-TDP2S60D fusion proteins were purified using a GST-fusion protein purification kit (Invitrogen) following the manufacturer's protocol.

*In vitro* phosphorylation assay was carried out in 40 mM Tris HCl (pH 7.5), 10 mM MgCl_2_, 0.1 mM EGTA, 1 mM dithiothreitol, and 5 mM β-glycero-phosphate. Each reaction contains 30 ng of purified ERK3 kinase and 1 μg of purified protein substrate, 5 μCi ^32^ P-ATP (Perkin Elmer), 25 μM cold ATP in a total volume of 30 μl. The reaction was carried out at 30°C for 30 min and then stopped by adding 10 μl of 4 X SDS sample buffer. Proteins were resolved by SDS-PAGE gel, stained with Coomassie Brilliant Blue (Bio-Rad), and visualized by autoradiography.

### Generation of lung cancer cell pools stably expressing shRNAs by lentiviral transduction

Production of pseudolentiviral particles and generation of stable cell pools by lentiviral transduction were performed by following the manufacturer's instructions (Open Biosystems). Pseudolentiviruses were produced in TLA-293T cells by co-transfecting pGIPZ lentiviral shRNAmir expression construct and Trans-Lentiviral packaging plasmid mix. Pseudoviral particles were harvested 48 hrs post-transfection and concentrated using PEG-it virus precipitation solution (System Biosciences) by following the manufacturer's instructions. Cells were transduced with prepared virus with the addition of polybrene (5μg/ml). Two days post-transduction, cells were split and selected by puromycin (1 μg/ml) for 10 days. Knockdown of the targeted genes’ expression was verified by Western blotting analysis.

### Generation of lentiviruses expressing TDP2 cDNAs

Pseudotype lentiviruses were produced in 293T cells by cotransfecting lentiviral pCDH-TDP2 expressing construct and pPACK Packaging Plasmid Mix (System Biosciences), following the manufacturer's instructions. Pseudoviral particles were harvested 48 hours after transfection and concentrated using PEG-it Virus Precipitation Solution.

### Cell growth-drug sensitivity assay

Lung cancer cell pools stably expressing shGIPZ or shERK3 were seeded in 96 well plate. Twenty-four hour post seeding, cells were transfected either with non-targeting siRNA (siCtrl) or siRNA specifically targeting TDP2 (siTDP2). Two days post-transfection, cells were treated with etoposide at different concentrations (0.5 μM to 25 μM) for two days. Cell proliferation was determined using the CellTiter 96 AQueous MTS One Solution Cell Proliferation Assay Kit (Promega), following the manufacturer's instructions.

### 5′-Tyrosyl DNA phosphodiesterase activity assay

5′-Tyrosyl DNA phosphodiesterase activity assay was performed following the procedures reported previously [3] with minor modifications. TDP2 substrate, a 19-bp oligonucleotide linked with a phosphotyrosine at 5′ and labelled with FITC at 3′ (5′-Y-TCCGTTGAAGCCTGCTTTC-FITC-′), was synthesized by Midland Certified Reagent Company. 5′-Tyrosyl DNA phosphodiesterase activity assay was carried out at 30°C for 30 min by incubating the indicted amount of purified GST-TDP2 protein or 4 μl whole cell lysate (2 μg/ μl) with 200 nM of oligonucleotide substrate in a 20-μl reaction mix containing 100 mM NaCl, 20 mM Tris, pH 7.5, and 2 mM MgCl2 and 1mM DTT. For the reactions using whole cell lysate, 50 μM competitor single-stranded oligonucleotide (5′-CTAACTTGAGCGAAACGGT-′) was added to reduce nonspecific nucleolytic degradation of the duplex substrate. To determine the effect of ERK3 on TDP2 activity, 20 ng of purified active or heat inactivated (at 90°C for 30min) ERK3 protein was incubated with TDP2 at 30°C for 30 min in the presence of 30μM ATP before the oligonucleotide substrate was added. Reactions were stopped by adding a four-fold excess of Novex TBE-Urea loading buffer (Invitrogen). Reactions were resolved on Novex 15% TBE-Urea PAGE gels (Invitrogen). The FITC-labelled oligonucleotides were imaged by Fuji Gel doc system.

### Statistical analysis

Results are expressed as mean ± s.e. Statistical significance was determined by a two-sided Student's *t* test. A *P*-*value* less than 0.05 is considered statistically significant.

## SUPPLEMENTARY MATERIAL TABLE AND FIGURE


