# Supplementary material for: ERK3 regulates TDP2-mediated DNA damage response and chemoresistance in lung cancer cells
Source: Oncotarget. 2015 Dec 19;7(6):6665–75. doi: 10.18632/oncotarget.6682 (PMC4872741; doi:10.18632/oncotarget.6682)
Supplement: Supplementary file 1 [file oncotarget-07-6665-s001.pdf]

ERK3 regulates TDP2-mediated DNA damage response and chemoresistance in lung cancer cells

Supplementary Material

Supplemental Table 1 Identification of S60 of TDP2 as an ERK3 phosphorylation site. *In vitro* kinase reaction was performed by incubating a GST-TDP2 fusion protein with or without active ERK3 kinase. TDP2 phosphorylation was then analyzed by mass spectrometry. One phosphorylated peptide was identified in ERK3-treated TDP2 sample, whereas no phosphorylated peptides were identified in the control reaction lacking ERK3.

Peptide sequence	# Peptide Counts	Phosphorylation site
MERALNS*YFEPPVEE	10	S60

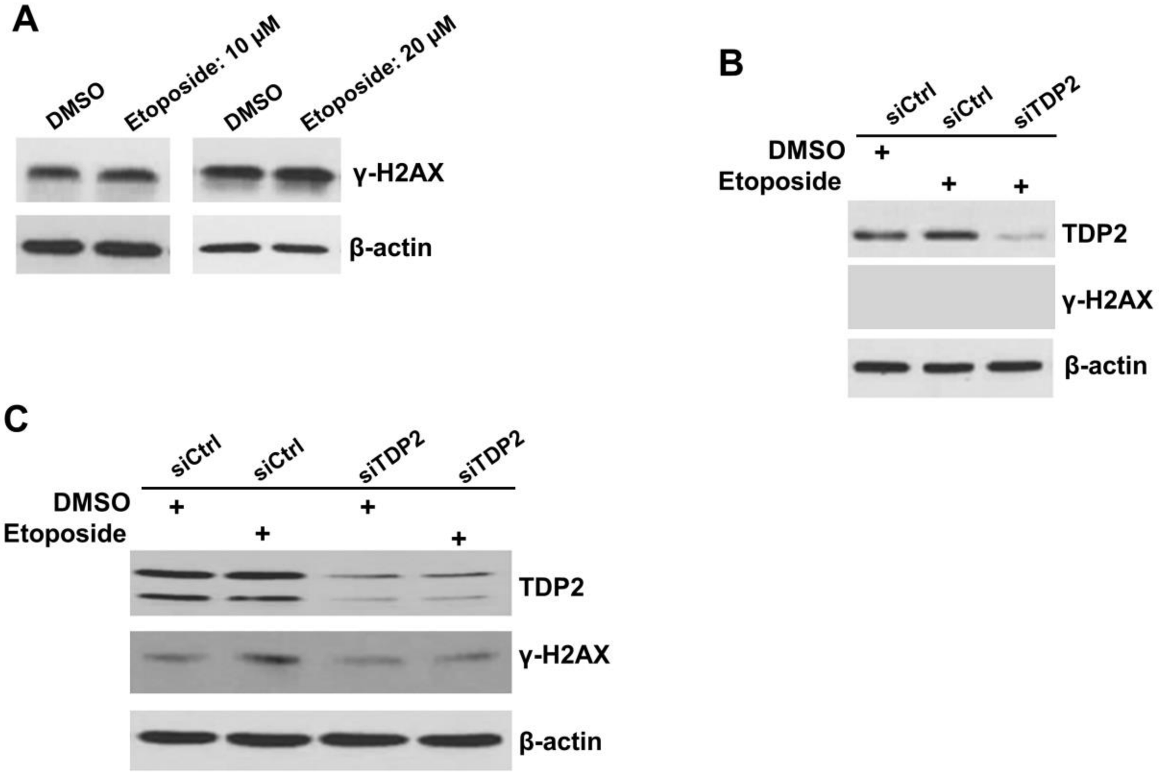

Figure S1 Lung cancer cell lines show differential response to the treatment of etoposide. (A). H157 cells were treated with etoposide or DMSO (vehicle control) for 1.5 hrs. Cells were then harvested and protein lysates were analysed by Western blotting using each specific antibody as indicated. **(B).** H1395 lung cancer cells were transfected with either siRNA against TDP2 (siTDP2) or non-targeting control siRNA (siCtrl). Two days after siRNA transfection, cells were treated with etoposide or DMSO for 1.5 hrs. γ-H2AX and TDP2 levels were analysed by Western blotting. **(C).** H1437 lung cancer cells were transfected with either siRNA against TDP2 (siTDP2) or non-targeting control siRNA (siCtrl). Two days after siRNA transfection, cells were treated with etoposide or DMSO for 1.5 hrs. γ-H2AX and TDP2 levels were analysed by Western blotting.
